# In Vivo Antibiotic Elution and Inflammatory Response During Two-Stage Total Knee Arthroplasty Revision: A Microdialysis Pilot Study

**DOI:** 10.3390/antibiotics14080742

**Published:** 2025-07-24

**Authors:** Julika Johanna Behrens, Alexander Franz, Frank Alexander Schildberg, Markus Rudowitz, Stefan Grote, Frank Sebastian Fröschen

**Affiliations:** 1 Department of Orthopedics and Trauma Surgery, University Hospital Bonn, 53127 Bonn, Germany; julika.behrens@ukbonn.de (J.J.B.); frank.schildberg@ukbonn.de (F.A.S.); frank.froeschen@ukbonn.de (F.S.F.); 2 Department of Trauma and Orthopedic Surgery, BG Klinik Ludwigshafen, 67071 Ludwigshafen, Germany; 3 Laboratory Dr. Wisplinghoff, 50858 Cologne, Germany; m.rudowitz@wisplinghoff.de; 4 Department of Orthopedics, Trauma and Hand Surgery, Barmherzige Brüder, Hospital St. Elisabeth, 94315 Straubing, Germany; stefan.grote@klinikum-straubing.de

**Keywords:** treatment monitoring, bone cement, minimal inhibitory concentration, PJI, arthroplasty, infection, two-stage exchange

## Abstract

**Introduction**: Two-stage revision with an antibiotic-loaded, temporary static cement spacer is a common treatment for periprosthetic joint infection (PJI) of the knee. However, limited data exists on in vivo antibiotic elution kinetics after spacer implantation. This pilot study uses the technique of microdialysis (MD) to collect intra-articular knee samples. The aim was to evaluate MD as an intra-articular sampling method to detect spacer-eluted antibiotics within 72 h after surgery and to determine whether they show specific elution kinetics. **Methods**: Ten patients (six male, four female; age median 71.5 years) undergoing two-stage revision for knee PJI were included. A MD catheter was inserted into the joint during explantation of the infected inlying implant and implantation of a custom-made static spacer coated with COPAL cement (0.5 g gentamicin (G) and 2 g vancomycin (V)). Over 72 h postoperatively, samples were collected and analyzed for spacer-eluted antibiotics, intravenously administered antibiotics (e.g., cefazolin and cefuroxime), metabolic markers (glucose and lactate), and Interleukin-6 (IL-6). Local and systemic levels were compared. **Results**: All catheters were positioned successfully and well tolerated for 72 h. Antibiotic concentrations in MD samples peaked within the first 24 h (G: median 9.55 µg/mL [95% CI: 0.4–17.36]; V: 37.57 µg/mL [95% CI: 3.26–81.6]) and decreased significantly over 72 h (for both *p* < 0.05, G: 4.27 µg/mL [95% CI: 2.26–7.2]; V: 9.69 µg/mL [95% CI: 3.86–24]). MD concentrations consistently exceeded blood levels (*p* < 0.05), while intravenously administered antibiotics showed higher blood concentrations. Glucose in MD samples decreased from 17.71 mg/dL to 0.89 mg/dL (*p* < 0.05). IL-6 and lactate concentrations showed no difference between MD and blood samples. **Conclusions**: Monitoring antibiotics eluted by a static spacer with intra-articular MD for 72 h is feasible. Gentamicin and vancomycin levels remained above the minimal inhibitory concentration. Differentiating infection from surgical response using metabolic and immunological markers remains challenging. Prolonged in vivo studies with MD are required to evaluate extended antibiotic release in two-stage exchanges.

## 1. Introduction

Periprosthetic joint infection (PJI) is a severe complication following total knee joint arthroplasty (TKA), often leading to revision surgery and prolonged antibiotic treatment. PJI incidence is approximately 1.4% to 1.7% after primary TKA and 4.9% to 7.8% after revision arthroplasty [1,2]. With increasing life expectancy, numbers of primary TKAs are estimated to increase by 45% in Germany until 2040, inevitably accompanied by rising numbers in revision arthroplasty due to PJI [3].

Treatments for PJI aim at eradicating infection and maintaining limb function. They include debridement, antibiotics, and implant retention (DAIR) in the case of well-fixed implants and a short duration of symptoms or one- or two-stage exchange in the case of a chronic infection or loosened implant [4]. Currently, two-stage revision is the gold standard for treating chronic PJIs [5,6]. Operative steps include complete removal of the infected implant, debridement of the infected soft tissue, irrigation, and implantation of a temporary custom-made cement prosthesis called a spacer [7]. The latter serves a dual function: one, by including antibiotics (e.g., gentamicin and vancomycin) within the used bone cement, it provides a high antibiotic concentration for local treatment while minimizing systemic side effects, and two, it maintains the size of the knee joint cavity [4,8]. In a second procedure, typically after six to eight weeks of beginning intravenously and followed by oral antibiotic therapy, the spacer is removed, debridement and irrigation are repeated, and a new knee prosthesis is implemented.

This two-stage revision approach has proven to be a successful treatment for PJI of the knee [5,9]. However, it presents a burden to both the patient and the health care system. Patients diagnosed with PJI experience a diminished quality of life, compounded by limited mobility of the affected joint, prolonged antibiotic treatment, extended hospitalization, and potential need for multiple surgical interventions [10,11]. Furthermore, individuals with a PJI face a significantly higher risk of morbidity when compared to those undergoing primary arthroplasty [12]. Moreover, treating PJI is becoming a socioeconomic burden for health care systems. Several studies have shown that revision arthroplasty for PJI is associated with severely high costs, with two-stage revision costing twice as much as DAIR for PJI after TKA [2,13,14].

The effectiveness of local antibiotic delivery by spacers when managing a PJI is an ongoing discussion [5], particularly concerning the elution kinetics from the spacer and sustaining therapeutic levels throughout implantation [8,15]. While systemic pharmacokinetics are well documented, local intra-articular antibiotic kinetics are less understood [8]. In this context, our preliminary study introduces microdialysis (MD) as a novel in vivo technique for real-time sampling directly from the knee joint. MD emerged as a promising minimally invasive technique for continuous measurement of unbound drug concentrations, such as antibiotics, in interstitial fluid of human tissue [16,17]. It quickly became a widely used tool in pharmacokinetic and pharmacodynamic research, in both animal models and clinical studies [18,19,20,21,22,23]. The technique operates on the principle of passive diffusion driven by concentration gradients across a semipermeable membrane within a probe [16,17]. This probe is inserted into the target tissue, and a physiological perfusion fluid is continuously pumped through it at low flow rates between 0.2 and 5 µL/min. As the perfusate flows, analytes of interest diffuse from the surrounding tissue into the probe. The resulting solution, known as dialysate, contains the recovered analytes and is collected in microvials for analysis.

With this pilot study, we want to evaluate the feasibility and diagnostic value of the MD technique for the first time in an in vivo PJI treatment to provide insights into postoperative pharmacokinetics of locally delivered antibiotics within the knee joint cavity. Therefore, the primary aim of this pilot study was to evaluate MD as an intra-articular sampling method, to detect spacer-released antibiotics within 72 h after surgery and to determine specific elution kinetics. Additionally, the MD technique was used to assess the metabolic and inflammatory constitution of the operated knee joint within the first three postoperative days.

## 2. Results

Demographic data, side of operated knee, number of previous PJIs, comorbidities, and cement mass used for the ten patients are represented in Table 1.

Three patients had no detectable pathogens in intraoperatively taken tissue samples. The other seven patients showed “common” organisms for PJI, including *Staphylococcus epidermidis*, *Staphylococcus aureus*, *Staphylococcus haemolyticus*, *Cutibacterium acnes*, and *Candida metamorphosis*. For intravenous antibiotic treatment, patients received either cefazolin, cefuroxime, clindamycin, or piperacillin and tazobactam, or a combination thereof. Three patients received vancomycin intravenously postoperatively, one as a preoperative treatment. We performed separate analyses regarding vancomycin concentrations (“vancomycin all” group, *n* = 10) and patients only receiving vancomycin through the spacer (“spacer only” group, *n* = 6). MD continuously generated intra-articular samples of the knee joint cavity, and no adverse effects (e.g., swelling, redness, pus, and warmth or pain at incision site) were observed.

### 2.1. Antibiotic Concentrations

**Figure 1:**
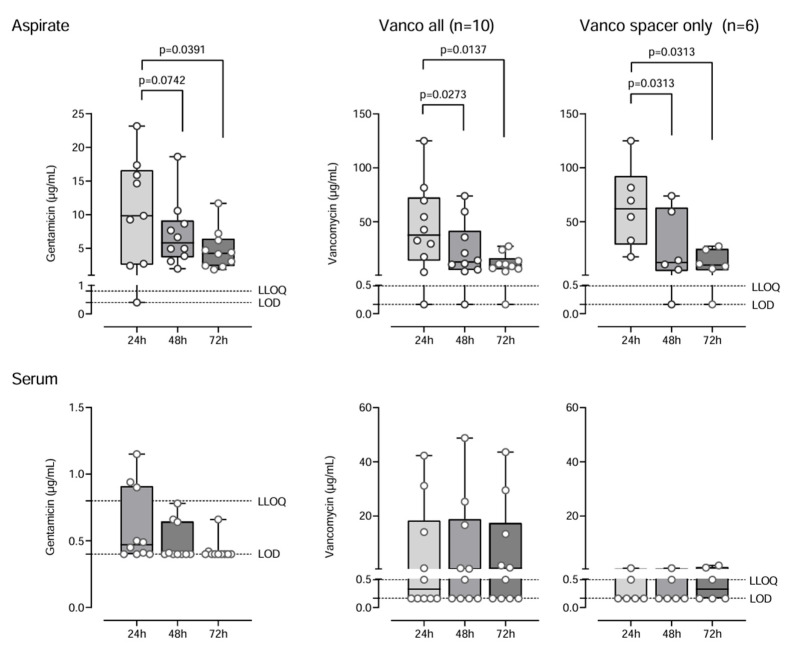
Gentamicin and vancomycin concentrations in microdialysis and blood samples. Gentamicin and vancomycin concentrations in knee microdialysis samples (aspirate, above) and blood samples (serum, below) for 72 h postoperative. Data are portrayed as median with the Interquartile Range as boxes and the minimum and maximum as whiskers. Lower limit of detection (LOD) and lower limit of quantification (LLOQ) are specific for each antibiotic and analytical method: gentamicin LOD: 0.4 µg/mL, LLOQ: 0.8 µg/mL; vancomycin LOD: 0.163 µg/mL, LLOQ: 0.489 µg/mL.

During the observation period, vancomycin and gentamicin displayed comparable kinetics in MD samples with initial high concentrations after 24 h (median gentamicin 9.55 µg/mL [95% CI: 0.4–17.36]; vancomycin all 37.57 µg/mL [95% CI: 3.26–81.6]; vancomycin spacer only 62.06 µg/mL [95% CI: 17.4–125]) followed by a significant decrease in concentrations within 72 h by 1.7-fold and 2.5-fold for gentamicin and 1.9-fold and 4.1-fold for vancomycin, respectively (gentamicin 4.27 µg/mL [95% CI: 2.26; 7.2]; vancomycin all 9.69 µg/mL [95% CI: 3.86–24]; vancomycin spacer only 9.79 [95% CI: 0.163–27.2]). For both antibiotics, the reduction in concentration in MD samples from 24 h to 72 h was significant with *p* < 0.05 (Figure 1). Inter-individual variability in antibiotic concentrations was high. Gentamicin and vancomycin (spacer only) concentrations were significantly higher in MD compared to blood serum samples (*p* < 0.05).

A significant positive correlation was observed between cement mass and intra-articular antibiotic levels (gentamicin r = 0.4863, vancomycin r = 0.4771).

Systemically administered antibiotics were detected in both compartments with consistently higher and effective but not quite significantly different levels in blood samples (*p* > 0.05).

### 2.2. Metabolic and Inflammatory Markers

Glucose levels dropped significantly over time from 17.71 mmol/L (95% CI: 5.59–63.03) at 24 h to 0.89 mmol/L (95% CI: 0.14–20.14) after 72 h (*p* < 0.05). There was no significant variation in lactate concentrations, with median levels between 5.11 mmol/L and 7.11 mmol/L.

For Interleukin-6 (IL-6), there was no statistically significant difference between serum and MD concentrations. We observed a trend towards decreasing IL-6 concentrations within 72 h in the blood and knee MD (Table 2).

## 3. Discussion

This pilot trial was able to successfully monitor local antibiotic therapy continuously in PJI therapy by implementing an MD catheter for the first time in the knee joint. Initial spacer-eluted antibiotic concentrations were high, followed by a consistent decline over 72 h postoperatively. Gentamicin and vancomycin concentrations were significantly higher in knee MD compared to serum samples. Further, metabolic markers (lactate and glucose) and IL-6 were detected in intra-articular samples.

### 3.1. Microdialysis

MD enables continuous measurement of unbound, pharmacologically active antibiotic concentrations at the target site, which may significantly differ from plasma levels due to local distributional barriers and tissue-specific kinetics [16,24]. While this method has been widely applied in neurological and soft tissue contexts, its application in monitoring antibiotic elution from static knee spacers remains underexplored. Other studies have already used MD for intra-articular antibiotic sampling in porcine models [23,25,26]. To the best of the authors’ knowledge, this has not been transferred to a human model so far. In this pilot study, we demonstrate the feasibility and safety of intra-articular MD for tracking gentamicin and vancomycin concentrations over 72 h in vivo, reproducing elution patterns observed in previous reports.

The semipermeable membrane of the MD probe serves as a selective barrier, preventing large molecules and bacteria from crossing into the perfusate, thereby minimizing infection risk during sampling [24]. However, a key limitation of the technique is that the collected dialysate represents only a fraction of the actual extracellular concentration. This “relative recovery” is highly influenced by the perfusate flow rate: higher flow rates (e.g., 2–5 µL/min) reduce recovery due to insufficient equilibration, whereas lower flow rates (e.g., 0.2–0.5 µL/min) provide better approximation of true tissue concentrations but require longer collection periods and reduce temporal resolution [16,24]. Given the novelty of the sampling site and analytical process, we opted for a flow rate of 2 µL/min to ensure sufficient sample volume for analysis [24,27].

### 3.2. Antibiotics

To the best of the authors’ knowledge, there is no current guideline regarding the concentration or type of cement or its preparation when treating PJI of the hip or knee. There are multiple in vitro studies investigating various conditions for antibiotic elution of spacers [28,29,30]. Our results are well in line with these in vitro studies, showing initially high antibiotic concentrations followed by a rapid decline within three days [29,31,32]. The transfer of in vitro results to an in vivo situation should be carried out cautiously since in vivo a spacer is exposed to different conditions (vascularized tissue, joint fluid variability, and varying cement surface area) [29].

### 3.3. Antibiotic Elution Kinetics and Therapeutic Effects

Antibiotic elution from the spacer has been described as a process of diffusion, enabling joint fluid to release antibiotics through the washout process [33]. Therefore, the surface area of the spacer and amount of surrounding joint fluid are critical factors influencing the release kinetics, next to the dosage and quantity of antibiotics and the type of bone cement and its porosity and preparation process [8]. All these varying factors explain the observed high inter-personal variability of gentamicin and vancomycin concentrations in intra-articular samples (Figure 1).

The initial burst is caused by a surface phenomenon, eluting antibiotics from outer cement layers followed by a sustained release phase, which is driven by elution from deeper zones based on the slow perfusion of joint fluid or hematoma through the cement [34]. This sustained release phase is attributed to being the primary reason for the prolonged maintenance of lower antibiotic concentrations [34]. Mutimer et al. analyzed intra-articular gentamicin levels in a synovial knee MD during spacer explantation surgery after a mean implantation period of 99 days and detected a median concentration of 0.46 mg/L towards the end [35]. This indicates that antibiotic concentrations were still detectable after about three months, suggesting a functional release for a prolonged time and consistent with other studies [36,37]. However, the amount of antibiotics must be put into context of their therapeutic levels and, therefore, effectiveness.

In this study, the median concentrations measured on day three in MD samples were eight times greater for gentamicin and five times greater for vancomycin than the minimal inhibitory concentrations. Therapeutic levels for vancomycin in the blood were between 15 and 20 mg/L and for gentamicin between 5 and 8 µg/mL [38,39]. Applying these values to intra-articularly taken samples for our study, therapeutic target concentrations for gentamicin were reached in all patients and in five patients for vancomycin. Serum levels remained subtherapeutic unless vancomycin was given systemically. This supports the concept that local delivery is effective in achieving high site-specific concentrations, with minimal systemic exposure. Only systemically administered antibiotics had higher and effective levels in blood samples.

### 3.4. Immunological and Metabolic Parameters

Several parameters have been discussed as diagnostic criteria for PJI, including glucose, lactate, and IL-6 [40,41,42]. As PJI includes the adherence of bacteria to the prosthesis or surrounding tissue, glucose used for metabolic consumption by bacteria can be inversely related to infection [43]. The normal glucose level in synovial fluid is within 0.56 mmol/L of the blood glucose level [44]. Kinugasa et al. suggests that a joint fluid level of glucose less than 2.22 mmol/L indicates pathogens [45]. Most patients had intra-articular glucose below the 2.22 mmol/L threshold, supporting a potential bacterial presence.

Elevated lactate concentrations have been reported during infection as a byproduct of anaerobic metabolism in bacteria [46]. Studies have shown lactate as being a promising marker for differentiating between septic and non-septic arthritis, with cut-off values between 6.95 mmol/L and 7.5 mmol/L [40,41,47]. From the current study, five of the patients were above 6.95 mmol/L for at least three of the six assessed time points, indicating bacterial activity. Elevated lactate concentrations could also be explained by hypo-perfused tissue during inflammation of PJI, leading to an analogous increase in anaerobic metabolism or as a result of surgical intervention [46].

As a pro-inflammatory cytokine, IL-6 is released by immune cells (e.g., macrophages and T cells) in response to bacterial infection to initiate C-reactive protein production, making it a sensitive marker to monitor inflammation [42,48]. In a meta-analysis, Xie et al. concluded that, while IL-6 demonstrates a high diagnostic value for PJI, no definitive cut-off values have been established, with reported values ranging from 359.3 pg/mL to 13.35 pg/mL, all of which considerably exceed the synovial IL-6 levels observed in our study [42]. Increases in IL-6 serum concentrations can be linked to tissue injury and surgery [49,50]. Therefore, elevated lactate concentrations within the first 24 h are more likely caused by local surgical reactions than a bacterial infection.

## 4. Materials and Methods

### 4.1. Study Design

This diagnostic–interventional pilot study was part of the routine treatment of 10 patients with a PJI of the knee at the Department of Orthopedics and Trauma Surgery at the University Hospital Bonn between January and June 2024. This study was approved by the local ethics committee of the University Hospital Bonn (local review board number 334/23-EP). Participation required patients to have MSIS-confirmed PJI and approval for surgery by the orthopedic and anesthesiologic departments [51]. Furthermore, patients had to be over 18 years of age and had to give written, informed consent. Patients with known allergies to gentamicin and vancomycin were excluded from the study.

Local (gentamicin and vancomycin) and systemic (e.g., cefazolin and cefuroxime) antibiotic concentrations were measured in intra-articular and systemic blood samples for 72 h postoperatively. To further characterize the inflammatory response within the knee joint, IL-6 as an immunological marker was measured in both compartments along with metabolic factors (lactate and glucose).

### 4.2. Intraoperative Procedures

**Figure 2:**
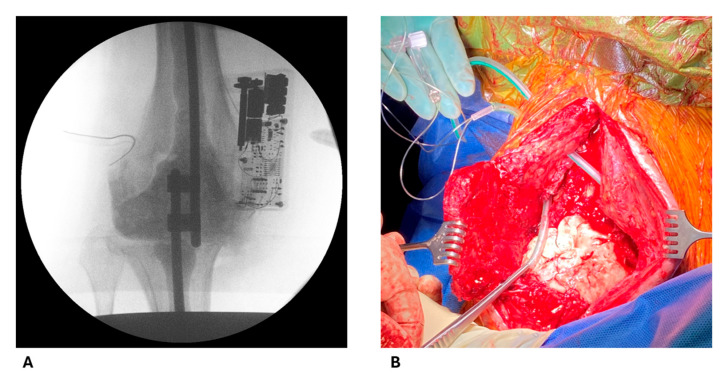
Spacer and catheter placement. (**A**) Postoperative X-ray photograph showing the metal rods implanted into the tibia and femur and connected at the previous knee joint line. The redon tube with the microdialysis catheter is seen on the left and the pump connected to the catheter on the right. (**B**) The modeling of the spacer has been concluded. The drainage tube containing the microdialysis catheter has been inserted in the lateral compartment of the knee joint (tube held with forceps) and will be placed laterally on the cement. The inlet and outlet tube (held in hand) will be connected accordingly to the microdialysis pump and microvial. The second drainage tube located further cranial is used as a redon drain.

After removing the infected implant, tissue samples from bone and soft tissue were taken for bacterial culture. The custom-made static spacer, consisting of two 6 mm titan rods (Cempadic R 6 × 400 mm, Implantcast, Buxtehude, Germany), was coated with COPAL gentamicin- and vancomycin-loaded bone cement (Heraeus Medical, Wehrheim, Germany). The rods were inserted into the tibial and femoral medullary canals and fixated with connectors at the overlapping ends (Figure 2A). One package of 43 g acrylic bone cement contained 0.5 g gentamicin and 2 g vancomycin. The preparation of the bone cement followed the manufacturer’s instructions. The bone cement was prepared in non-vacuum conditions, and antibiotics were commercially mixed within the cement. Further bone cement preparation depended on joint cavity size, with additional cement used as needed (Figure 2B).

**Figure 3:**
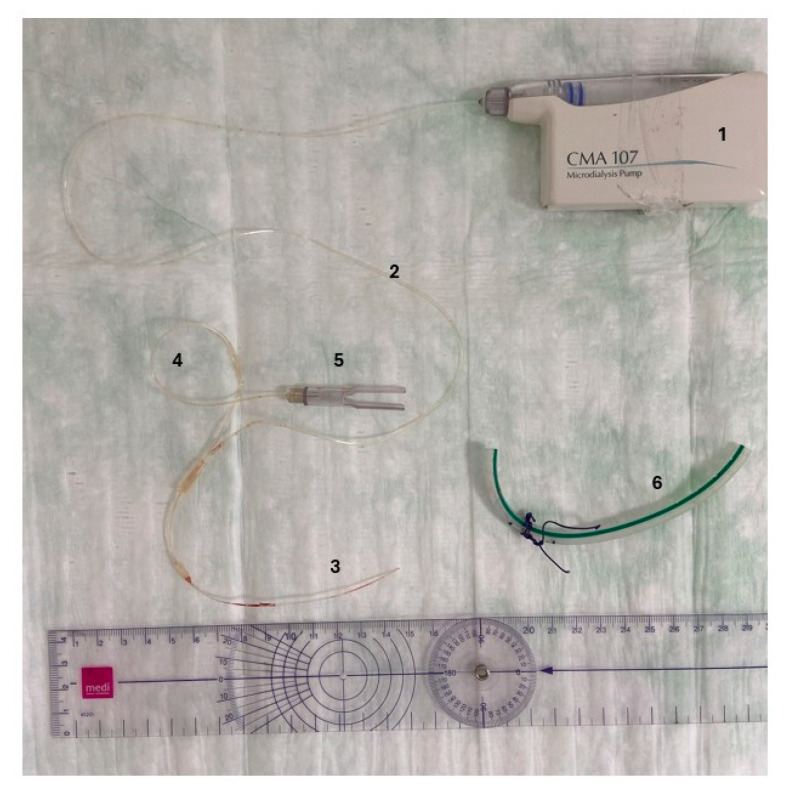
Microdialysis catheter. The microdialysis catheter is connected to its pump (1) shown next to a drainage tube (6). The syringe is already inserted into the pump from where the inlet tube (2) continues to the dialysis membrane (3). The outlet tube (4) connects to the microvial holder (5). The drainage tube (6) was used to insert the catheter.

The MD catheter (71 High Cut-Off, M Dialysis AB, Stockholm, Sweden) was inserted into the knee joint cavity post-polymerization (Figure 2B). For protection of its sensitive membrane and to ensure complete removal, it was inserted through a regular 16-gauge wound drainage tube (B. Braun SE, Melsungen, Germany). The MDs design and drainage tube can be seen in Figure 3. Placement of the drainage tube was standardized laterally to the cavity filling cement. The catheter allowed for the diffusion of particles up to 100,000 Dalton along a semipermeable membrane due to a perfusion flow maintained by the 107 Microdialysis Pump (M Dialysis AB, Stockholm, Sweden). Samples were collected in microvials (M Dialysis AB, Stockholm, Sweden).

### 4.3. Sample Collection Protocol

MD sample collection was conducted over 72 h at 0.5 µL/min and 2 µL/min flow. Samples were pooled every 24 h for pharmacological and immunological analysis. Two vials per interval were reserved for metabolic analysis. Venous blood samples were drawn daily before antibiotic administration to ensure trough levels.

### 4.4. Analysis

Intra-articular samples in microvials were stored in a fridge (5–8 °C) during collection and after aliquoting were stored frozen at −80 °C alongside blood serum samples after centrifugation for further analysis. Antibiotic concentrations and IL-6 were analyzed in both MD and blood samples, whereas metabolic parameters were only analyzed in MD samples.

To determine the concentration of gentamycin in serum and MD samples, an automated immunoassay based on the kinetic interaction of microparticles in solution (KIMS) was used, utilizing GENT2 ONLINE TDM Gentamicin and a Cobas^®^ c 703 (Roche Diagnostics, Mannheim, Germany). Other antibiotics were measured via liquid chromatography–tandem mass spectrometry (LC-MS/MS) using the ClinMass^®^ TDM Kit System by RECIPE (RECIPE Chemicals + Instruments, Munich, Germany).

IL-6 concentrations were determined with an enzyme-linked immunosorbent assay (ELISA, Human IL-6 DuoSet, Bio-Techne, Minneapolis, MN, USA) and metabolic parameters with an ISCUSflex Microdialysis Analyser (M Dialysis AB, Stockholm, Sweden).

Pathogen identification was performed using matrix-assisted laser desorption/ionization time-of-flight (MALDI-TOF) mass spectrometry (bioMérieux, Nürtingen, Germany). Antimicrobial susceptibility testing was primarily conducted using the automated Vitek2 system (bioMérieux, Nürtingen, Germany). For anaerobic organisms, susceptibility testing was conducted using a semi-automated microtiter broth dilution method (MICRONAUT, Merlin, Bornheim, Germany). Interpretation of susceptibility results followed the EUCAST clinical breakpoints, version 13.1 (2023).

### 4.5. Statistics

Data were collected in Microsoft Excel 2024 (Microsoft Corporation, Richmond, VA, USA) and analyzed using GraphPad Prism 9.1.2 (GraphPad Software, Boston, MA, USA). All data are reported as median values with 95% confidence intervals. The Wilcoxon matched pair signed rank test was used for comparison, with *p* < 0.05 considered significant.

## 5. Conclusions

In this pilot study, we demonstrate the feasibility and safety of intra-articular MD for continuous monitoring of spacer-derived antibiotic concentrations, gentamicin and vancomycin, over 72 h, reproducing elution patterns observed in previous reports. Metabolic and inflammatory markers (glucose, lactate, and IL-6) provided supplementary insights into joint environment changes, though their specificity remains limited in the early postoperative period.

This study provides foundational data on local antibiotic kinetics following spacer implantation, supporting future efforts to evaluate therapeutic success and defining appropriate spacer duration based on antibiotic elution. By using this technology and the growing information regarding local antibiotic therapy and inflammation monitoring, MD application could be used in the future to individualize therapy durations, determine optimal timing for prosthesis reimplantation, thereby reducing hospital costs, and ultimately optimize PJI treatment.

### Limitations

A small sample size of ten multimorbid patients, along with the heterogeneity of parenterally applied antibiotics and detected microbiological pathogens, made a comparison across the cohort and the generalization of our findings difficult. The short observation period limits the insight into long-term intra-articular antibiotic elution by the spacer. Furthermore, this study lacks a control group, and we focused on a single type of spacer, always including gentamicin and vancomycin but varying cement amounts. While these factors restrict broader generalization, we believe that for a proof-of-concept study of the applied MD technique, the cohort is sufficient.

Additionally, several limitations intrinsic to MD may have affected the accuracy of the measured concentrations. These include the absence of probe calibration (e.g., retrodialysis), the usage of varying perfusate flow rates, and possible variation in diffusion kinetics due to factors such as varying amounts of joint fluid or postoperative hematoma surrounding the catheter. Our used, comparatively high perfusion flows tend to underestimate the target site concentrations. Preanalytical factors, such as storage conditions including temperature fluctuations (during sample generation next to the patient, storage at 5 °C before conglomeration, storage at −80 °C until analysis), may also contribute to the degradation or adsorption of targeted molecules.

Regarding antibiotic analysis, LC-MS/MS represents a sensitive method where late-phase (72 h) or low-exposure (systemic antibiotics in MD) samples lead to uncertainty in result interpretation when falling close or below the LLOQ. Regarding our analysis, when comparing IL-6 values, methods of analysis have to be considered as reasons for possible differences between studies.

Future studies aiming for more accurate intra-articular pharmacokinetic profiling should consider lower perfusate flow rates, along with appropriate calibration techniques (e.g., retrodialysis or other in vivo approaches), to improve quantification and resolution. Furthermore, investigating a larger cohort with multiple spacer types including different antibiotics and preparation procedures (e.g., preformed spacers) should be over a prolonged period of time (e.g., 10 days) to follow the displayed elution kinetics and improve clinical applicability.

## Figures and Tables

**Table 1:** Demographic data and patient comorbidities.

Number of Patients	*n* = 10	
Gender	6 Male	
	4 Female	
Age (years)	71.5	[67–78]
BMI (kg/m^2^)	36.91	[35.11–42.68]
Operated limb	7 right	
	3 left	
Amount of prepared cement (g)	90	[63–132]
Comorbidities		
Arterial hypertension	8	
Type 2 diabetes	5	
Cardiac insufficiency	5	
Adipositas (Grade I/II/III)	7	(1/3/3)
Previous PJI	5	

Data presented as median and 95% CI in square brackets.

**Table 2:** Concentrations of Interleukin-6 (IL-6) in blood serum and knee aspirate samples.

		24 h		48 h		72 h	
		Median	95% CI	Median	95% CI	Median	95% CI
IL-6 [pg/mL]	serum	24.83	[7.46–126.2]	17.18	[0–116.4]	11.85	[0–122.8]
	aspirate	73.99	[36.54–125.9]	27.71	[0–64.07]	17.74	[0–36.07]
	*p*-value	0.275		0.734		0.652	

Data displayed as median and 95% confidence interval. The *p*-value for serum and aspirate measurements was not significant for *p* > 0.05.

## Data Availability

The data supporting the findings of this study are available from the corresponding author upon reasonable request. Source data underlying all figures and tables are provided as a source.

## Abbreviations

The following abbreviations are used in this manuscript:

PJI	Periprosthetic joint infection
TKA	Total knee arthroplasty
DAIR	Debridement, antibiotics, and implant retention
MD	Microdialysis
IL-6	Interleukin-6
